# Treatment of non-small cell lung cancer with intensity-modulated radiation therapy in combination with cetuximab: the NEAR protocol (NCT00115518)

**DOI:** 10.1186/1471-2407-6-122

**Published:** 2006-05-08

**Authors:** AD Jensen, MW Münter, H Bischoff, R Haselmann, C Timke, R Krempien, F Sterzing, S Nill, S Heeger, A Hoess, U Haberkorn, PE Huber, M Steins, M Thomas, J Debus, KK Herfarth

**Affiliations:** 1 Dept. of Radiation Oncology, Clinical Radiology, University of Heidelberg Medical School, INF 400, 69120 Heidelberg, Germany; 2 Dept. of Medical Physics, German Cancer Research Centre (DKFZ), INF 280, 69120 Heidelberg, Germany; 3 Clinical Co-operation Unit Radiation Oncology, German Cancer Research Centre (DKFZ), INF 280, 69120 Heidelberg, Germany; 4 Dept. of Medical Oncology, Thoraxklinik Heidelberg, Amalienstr. 5, 69126 Heidelberg, Germany; 5 Dept. of Nuclear Medicine, University of Heidelberg Medical School, INF 400, 69120 Heidelberg, Germany; 6 Merck KGaA, Darmstadt, Germany

## Abstract

**Background:**

Even today, treatment of Stage III NSCLC still poses a serious challenge. So far, surgical resection is the treatment of choice. Patients whose tumour is not resectable or who are unfit to undergo surgery are usually referred to a combined radio-chemotherapy. However, combined radio-chemotherapeutic treatment is also associated with sometimes marked side effects but has been shown to be more efficient than radiation therapy alone.

Nevertheless, there is a significant subset of patients whose overall condition does not permit administration of chemotherapy in a combined-modality treatment.

It could be demonstrated though, that NSCLCs often exhibit over-expression of EGF-receptors hence providing an excellent target for the monoclonal EGFR-antagonist cetuximab (Erbitux^®^) which has already been shown to be effective in colorectal as well as head-and-neck tumours with comparatively mild side-effects.

**Methods/design:**

The NEAR trial is a prospective phase II feasibility study combining a monoclonal EGF-receptor antibody with loco-regional irradiation in patients with stage III NSCLC. This trial aims at testing the combination's efficacy and rate of development of distant metastases with an accrual of 30 patients.

Patients receive weekly infusions of cetuximab (Erbitux^®^) plus loco-regional radiation therapy as intensity-modulated radiation therapy. After conclusion of radiation treatment patients continue to receive weekly cetuximab for 13 more cycles.

**Discussion:**

The primary objective of the NEAR trial is to evaluate toxicities and feasibility of the combined treatment with cetuximab (Erbitux^®^) and IMRT loco-regional irradiation.

Secondary objectives are remission rates, 3-year-survival and local/systemic progression-free survival.

## Background

80% of all lung cancers are non small cell carcinomas. For these tumours, complete surgical resection still yields the best treatment results so far. However, only 25% of all patients have the option of surgical treatment.

In the event of the tumour being surgically not resectable or the patient functionally inoperable, radiation therapy/combined radio-chemotherapy are the only curative treatment options for lung cancer in a localised stage. In this case, a dose of 60–66 Gy is usually applied to the tumour by external beam radiotherapy (EBRT) resulting in a mean local tumour control of about 12 months [1]. Furthermore, a recent meta-analysis was able to demonstrate improved results in combined radio-chemotherapy on platinum-based regimen with a significantly higher 2-year-survival compared to local irradiation alone [2]. It could also be shown in various randomised trials that simultaneous platinum-based radio-chemotherapy is significantly superior to sequential regimen [3-5]. Accompanying toxicities are, however, not negligible, especially considering the simultaneous radio-chemotherapy [3] which is the reason for many patients proving ineligible for a combined treatment.

Other potential partners for combined treatment are monoclonal antibodies. NSCLCs often show an over-expression of epidermal growth factor receptors (EGFR) [6,7] also associated with a less favourable prognosis. In pre-clinical experiments EGFR inhibition was able to show a reduction of cell proliferation, an increase of apoptosis, and a reduction of angiogenesis [8,9].

Cetuximab is a monoclonal antibody which binds to the extracellular EGF-receptor domain hence inhibiting intracellular phosphorylation of EGFR and consecutive down stream signalling. This in turn causes cell cycle arrest and increased expression of pro-apoptotic enzymes.

Combining irradiation and cetuximab exposure, a synergistic and/or additive effect could be demonstrated in NSCLC cell lines in vitro [10].

In the case of squamous cell carcinoma of the head and neck, a G_0_/G_1_-cell cycle arrest could be observed with the radiation-induced damage exhibiting a reduction of repair and an increase in apoptosis compared to irradiation alone [9-11].

There are various phase I-III trials which were able to demonstrate that cetuximab can be safely administered as a single drug and also in combination with irradiation [14-19].

In a large phase III trial, patients with head and neck tumours were randomized either to irradiation alone or in combination with cetuximab. 424 patients were enrolled in this trial showing a significantly higher 3-year survival of 55% in the combined treatment vs. 45 % for irradiation alone [18]. These encouraging results show a good correlation to results obtained in combined radio-chemotherapy vs. irradiation alone in locally advanced head and neck cancer [20]. However, combining irradiation and cetuximab also resulted in an increase of skin reactions [18].

In conclusion, there are good reasons to expect improvement of treatment results with respect to local tumour control and acceptable toxicity on combining irradiation and application of EGF-receptor antibodies.

The main purpose of the NEAR-trial (**N**on-small cell lung cancer, **E**rbitux **A**nd **R**adiotherapy) is to evaluate the feasibility and safety of a new treatment regimen in inoperable NSCLC stage III by combining loco-regional irradiation and weekly application of the monoclonal EGFR- receptor antibody cetuximab (Erbitux^®^) in patients who are not eligible for a radio-chemotherapy.

## Methods/design

### Trial organization

NEAR has been designed by the Trial Center of the Department of Radiation Oncology, University of Heidelberg in cooperation with the Thoraxklinik in Heidelberg. The trial is carried out by the Department of Radiation Oncology together with the German Cancer Research Center (DKFZ) and Department of Medical Oncology of the Thoraxklinik Heidelberg. The trial is an investigator initiated trial. Trial medication (cetuximab) is supplied by Merck KGaA, Darmstadt, Germany.

### Coordination

The trial is co-ordinated by the Department of Radiation Oncology of the University of Heidelberg in cooperation with the DKFZ and the Department of Medical Oncology at the Thoraxklinik Heidelberg. The Dept. of Radiation Oncology is responsible for overall trial management, trial registration (ClinicalTrials.gov Identifier: NCT00115518), database management, quality assurance including monitoring, reporting and for the scientific program of all trial related meetings.

### Investigators

Patients will be recruited by the Department of Radiation Oncology at the University of Heidelberg and by the Department of Medical Oncology of the Thoraxklinik Heidelberg. Due to the multi-modal nature of the trial, all investigators are experienced oncologists from the fields of radiation oncology and medical oncology.

### Adverse events committee

This committee consists of 2 physicians (medical oncologist, radiation oncologist) and decides on the final diagnostic classification of critical clinical events. For all serious adverse events the documentation and relevant patient data are verified by the co-ordinating personnel before submitting the data to the Adverse Events Committee for diagnostic classification.

Analysis of safety related data is performed with respect to frequency of:

• Serious Adverse Events and Adverse Events stratified by organ-system

• Adverse Events stratified by severity

• Adverse Events stratified by causality.

Patient toxicities will be assessed using the NCI Common Toxicity Criteria (CTC). Toxicity will be evaluated prior to treatment, weekly prior to each course of infusional Cetuximab and at follow-up. Unacceptable toxicity is defined as unpredictable, or irreversible Grade 4 toxicity. Decisions regarding cetuximab dose-adjustment will be made using the guidelines below and based on haematological parameters (ANC and platelets) monitored weekly during radiation before each dose of cetuximab.

### On-site monitoring

During recruitment of patients monitoring on site is performed according to good clinical practice (GCP) guidelines. The data management will be performed by the Trial Centre of the Department of Radiation Oncology, University of Heidelberg.

### Ethics, informed consent and safety

The final protocol was approved by the ethics committee of the University of Heidelberg, Medical School (L-284/2004) and the Paul-Ehrlich-Institute ((PEI-registration number 1209/01). This study complies with the Helsinki Declaration in its recent German version, the Medical Association's professional code of conduct, the principles of Good Clinical Practice (GCP) guidelines and the Federal Data Protection Act. The trial will also be carried out in keeping with local legal and regulatory requirements. The medical secrecy and the Federal Data Protection Act will be followed.

Written informed consent is obtained from each patient in oral and written form before inclusion in the trial and the nature, scope, and possible consequences of the trial have been explained by a physician. The investigator will not undertake any measures specifically required only for the clinical trial until valid consent has been obtained.

### Study design

The NEAR-study is a prospective phase II feasibility study combining a monoclonal EGF-receptor antibody with loco-regional irradiation in patients with stage III NSCLC. This trial aims at testing the combination's safety and efficacy and rate of development of distant metastases with an accrual of 30 patients. These are treated by the Dept. of Radiation Oncology and Radiation Therapy, University of Heidelberg, in co-operation with the German Cancer Research Centre (DKFZ), Heidelberg, the Dept. of Medical Oncology, Thoraxklinik, Heidelberg, and the Dept. of Nuclear Medicine, University of Heidelberg.

Primary endpoints are toxicities and feasibility of the combined treatment.

Secondary endpoints are remission rates, 3-year-survival and local/systemic progression-free survival.

### Patient selection

In order to be included in the NEAR trial, patients were required to have histologically confirmed NSCLC and documented inoperable NSCLC stage III disease, where combined chemo-radiotherapy is either deemed medically contra-indicated or refused by the patient. Each patient is discussed by a commitee consisting of a thoracic surgeon, a medical oncologist, a pulmonologist, a radiologist and a radiation oncologist. Evaluation of EGFR status is recommended but not compulsory. Patients also need to have sufficient remaining lung function (FeV_1 _≥ 1.5 l/s or at least 50% of the respective individual norm value) as well as a Karnofsky Performance score of 70 % or higher.

Patients should be ≥ 18 years and individual life expectancy be estimated to ≥ 6 months. Accrued patients will be expected to demonstrate sufficient compliance, they should also live in relative proximity of the centre of care to ensure adequate follow-up after treatment. In addition, adequate haematological, hepatic, and renal function is essential for inclusion in the trial (wbc ≥ 3000 × 10^3 ^/ml, thc ≥ 100 × 10^6 ^/ml, hb ≥ 10 g/dl).

Female patients are required to use adequate contraception during and at least up to 3 months after treatment. Naturally, written informed consent is obtained prior to commencement of treatment in this trial.

Patients are not eligible with active infections, inadequate liver function (bilirubin ≥ 2 × above normal, GOT/GPT > 5 × above normal) or haemodynamically relevant haemoptyses (hb-drop of 1 g/dl within 24 h), superior vena cava syndrome, or malignant pleural effusions. Patients with severe concurrent systemic disease or other malignant disease (apart from cervical carcinoma in-situ, basal cell carcinoma, or unless previously treated and in remission for ≥ 5 years without further treatment) can also not be included in this trial. Furthermore, hypersensitivity to foreign proteins or x-ray contrast medium, or use of other test substances within one month prior to commencement of the NEAR trial and previous chemotherapy will prohibit inclusion as will pregnancy or breast feeding.

### Work-up

Patients with pathologically or cytologically documented NSCLC stage III receive a complete work-up including thoracic and cerebral CT scans, abdominal ultrasound, bone scan (plus x-ray exams of suspect areas where applicable). Patients with positive mediastinal lymph nodes, (either pathologically documented or found suspect in the CT-scan due to diameter >1.5 cm) will be sent for surgical consultation and evaluation of operability. Should operation be found impossible, in,-and exclusion criteria are examined. In the event of patients meeting the required inclusion criteria, information about participation in the study with possible risks and benefits is given to the patients and written informed consent is obtained. Patients can then be included in the study, documentation is provided by the study centre (Studienzentrale Klinische Radiologie, Abt. Strahlentherapie und Radioonkolgie, INF 400, 69120 Heidelberg).

After inclusion, patients are referred to FDG-PET scanning in order to optimise target volume definition. Each patient receives a radiation therapy planning CT-scan in individually-adjusted precision immobilisation devices.

In the event of patients declining treatment within the NEAR trial, irradiation alone is offered.

### Safety and discontinuation of treatment

Toxicities are classified by grade, type, duration, onset, and relationship to study treatment.

Treatment of cetuximab-induced adverse reactions is carried according to recommendations by the provider.

For grade 1 or 2 allergic reactions, a decrease of infusion rate for current and subsequent infusions is suggested. For ≥°3, persistent °1 or °2 allergic reactions despite reduction of infusion rate, it is recommended to discontinue treatment with cetuximab. Skin reactions in terms of acne-like rash after cetuximab are common. In patients with °3 acne-like rash cetuximab should be delayed for up to two subsequent infusions. Treatment also includes concomitant topical and/or oral antibiotics where necessary. Therapy can be resumed on resolution of the rash to <°2. Cetuximab needs to be delayed on a 2^nd ^or 3^rd ^occurrence of a °3 skin reaction for up to two consecutive cycles with dose reduction to 200 mg/m^2 ^or 150 mg/m^2 ^respectively. Any further occurrence of grade °3 acne-like rash will lead to discontinuation of cetuximab treatment.

### Drug supply

The monoclonal antibody cetuximab (Erbitux^®^) is provided by Merck KGaA, Darmstadt, Germany, and stored by the University Hospital Pharmacy, Heidelberg.

The respective cetuximab dose applied in this setting corresponds to the recommended and approved dosage tested in combination with irinotecan in metastatic colo-rectal carcinoma [19]. Cetuximab is given with a loading dose of 400 mg/m^2 ^as an intravenous infusion on day 1. Subsequently, the regular weekly dose during radiotherapy is 250 mg/m^2 ^on days 8, 15, 22, 29, 36, 43, and 50. After completion of irradiation, patients continue to receive 250 mg/m^2 ^weekly for another 13 weeks. Administration of cetuximab is discontinued if patients show local or systemic progression.

### Radiation therapy

Irradiation is applied as intensity-modulated radiation therapy (IMRT). The primary tumour and mediastinal lymph nodes receive a dose of 50 Gy in daily fractions of 2 Gy (Monday to Friday).

Primary tumour and involved lymph nodes are subsequently boosted to a total dose of 66 Gy in daily fractions of 2 Gy. Tolerances of thoracic organs at risk should not be exceeded.

**Figure 1:**
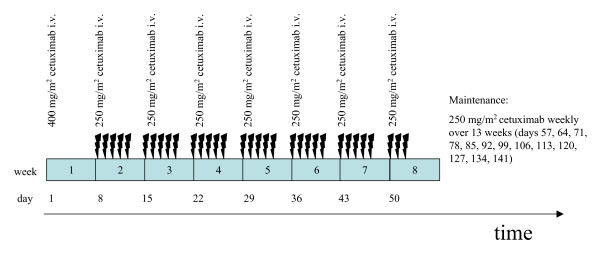
Chart of the treatment schedule of the NEAR trial.

The schedule of the treatment is shown in Figure 1:

### Supportive therapy

Whenever necessary, metoclopramide or 5-HT_3_-antagonists are used for antiemesis.

Antihistamines such as clemastine or dimetinden and steroids are administered prior to cetuximab-application. Skin reactions, especially acne-like rashes can be treated by topic or systemic antibiotics (i.e. tetracyclines or metronidazole) if necessary. Radiation induced skin reactions are treated according to in-house protocols with mild moisturizing lotion (i.e. Bepanthen^®^-lotio) or local application of steroids.

### Trial duration

Individual participation is completed either three years after enrolment or death of the patient.

Individual exclusion criteria are serious adverse reactions or the patient's voluntary withdrawal from the trial.

### Assessment of therapeutic efficacy

Methods/Investigations

• Chest x-ray on completion of irradiation to exclude disease progression under current treatment

• CT-thorax q3 months until completion of study

• Abdominal ultrasound q6 months until completion of study

• Bone scan q12 months until completion of study

• FDG-PET scan prior and after completion of the treatment (last cetuximab infusion)

### Evaluation

Local response is evaluated in accordance with the RECIST Criteria (Response Evaluation Criteria in Solid Tumours) [21].

1. complete remission (CR): is defined as complete regression of the treated tumour mass (confirmation after at least 4 weeks of treatment or later)

2. partial remission (PR) is defined as reduction of sum of largest tumour diameters by at least 30% (confirmation after at least 4 weeks of treatment or later)

3. stable disease (NC := no change): neither PR nor PD

4. progressive disease (PD): increase of sum of largest tumour diameters by 20%

## Discussion

Definitive radiation therapy as a single modality approach leads to a permanent cure only in a minority of patients with inoperable NSCLC stage III. Outcome in this group of patients was improved by administering concurrent radio-chemotherapy and combining local irradiation with various systemic platinum-based chemotherapy regimens. However, toxicities also increase in the combined modality approach so a major part of the respective patients are unfit to receive this combined treatment. One possible alternative could be the combination of irradiation with an EGF-receptor antibody like cetuximab. A phase III trial in head and neck tumours was already able to demonstrate significantly improved tumour response and overall survival on combining local irradiation with cetuximab vs. irradiation alone. These results were even comparable to standard radio-chemotherapy regimen in this setting.

Considering previous results with cetuximab by other investigators, there is a legitimate hope that side-effects can be minimised by a combined treatment of irradiation and cetuximab and will be mild in comparison to the standard radio-chemotherapy regimen. Also, by IMRT-treatment planning, normal tissue will be more adequately spared while allowing dose escalation to the gross tumour volume. Thus, this treatment might become an option even for patients otherwise unfit to receive the standard combined radio-chemotherapy regimen.

The NEAR trial was designed to evaluate the feasibility and toxicity of a combined treatment with irradiation and a standardised dosage of the monoclonal EGFR antibody cetuximab (Erbitux^®^) in inoperable NSCLC stage III.

## Competing interests

SH is employed by Merck KgaA (Darmstadt, Germany). JD is member of the advisory board 'head and neck tumours' of Merck KgaA.

## Authors' contributions

KKH, MWM, SH, and JD planned, co-ordinated and conducted the study. Medical care is covered by ADJ, CT, FS, MWM, MT, HB, MS and KKH. MWM, MT, HB, RH and KKH are responsible for patient recruitment. KKH, MWM, SN, CT, ADJ, FS, RH are taking part in conducting the trial. The scientific program was planned is carried out by KKH and MWM and UH SN and AH provided technical assistance and quality control in radiation treatment planning and delivery. All authors read and approved the final manuscript.

## Pre-publication history

The pre-publication history for this paper can be accessed here:
